# Secreted Factors from Colorectal and Prostate Cancer Cells Skew the Immune Response in Opposite Directions

**DOI:** 10.1038/srep15651

**Published:** 2015-10-27

**Authors:** Marie Lundholm, Christina Hägglöf, Maria L. Wikberg, Pär Stattin, Lars Egevad, Anders Bergh, Pernilla Wikström, Richard Palmqvist, Sofia Edin

**Affiliations:** 1 Department of Medical Biosciences, Pathology, Umeå University, Umeå, Sweden.; 2 Department of Surgical and Perioperative Sciences, Urology and Andrology, Umeå University, Umeå, Sweden.; 3 Department of Oncology-Pathology, Karolinska Institute, Stockholm, Sweden.

## Abstract

Macrophage infiltration has been associated with an improved prognosis in patients with colorectal cancer (CRC), but a poor prognosis in prostate cancer (PC) patients. In this study, the distribution and prognostic value of proinflammatory M1 macrophages (NOS2^+^) and immunosuppressive M2 macrophages (CD163^+^) was evaluated in a cohort of 234 PC patients. We found that macrophages infiltrating PC were mainly of an M2 type and correlated with a more aggressive tumor and poor patient prognosis. Furthermore, the M1/M2 ratio was significantly decreased in PC compared to CRC. Using *in vitro* cell culture experiments, we could show that factors secreted from CRC and PC cells induced macrophages of a proinflammatory or immunosuppressive phenotype, respectively. These macrophages differentially affected autologous T lymphocyte proliferation and activation. Consistent with this, CRC specimens were found to have higher degrees of infiltrating T-helper 1 cells and active cytotoxic T lymphocytes, while PC specimens displayed functionally inactive T cells. In conclusion, our results imply that tumour-secreted factors from cancers of different origin can drive macrophage differentiation in opposite directions and thereby regulate the organization of the anti-tumour immune response. Our findings suggest that reprogramming of macrophages could be an important tool in the development of new immunotherapeutic strategies.

Colorectal cancer (CRC) and prostate cancer (PC) are diseases of different organs where the immune response may have contrasting effects on patient prognosis. We have previously shown that in CRC, increased infiltration of macrophages is strongly associated with an improved prognosis^1^,^2^, while the opposite is true in PC^3^ and many other human cancers^4^.

The immune context has many important roles in tumour progression and patient prognosis^5^. For instance, the different subsets of macrophages and lymphocytes have unique and sometimes opposite effects. On the one hand, the acute inflammatory response is dominated by activation of M1 macrophages with important functions in antigen presentation and secretion of proinflammatory cytokines (e.g. IL1B, IL6, IL12, and TNF) and chemokines (e.g. CCL5, CCL7, CXCL9, CXCL10 and CXCL16) that regulate the recruitment and activation of T-helper 1 (Th1) and cytotoxic T lymphocytes (CTLs)^5^,^6^,^7^. The acute inflammatory response has tumouricidal activity, and is a central part of the host defense. On the other hand, if an acute inflammatory reaction cannot be resolved it may become chronic. Chronic inflammation is linked to immune suppression, which is in turn an important mechanism of tumour immune escape. Chronic inflammation is dominated by M2 macrophages, wound healing, phagocytosis and secretion of immunosuppressive cytokines (e.g. TGFB and IL10) and chemokines (e.g. CCL17, CCL22 and CCL24) resulting in the recruitment and polarization of T-helper 2 (Th2) and regulatory T lymphocytes (Tregs) that inhibit the activity of CTLs^5^,^6^,^7^. Macrophages are highly plastic cells that quickly respond to surrounding stimuli, and they can adopt many different shapes and functions^6^,^8^. In cancer, the macrophage phenotype is controlled by factors in the microenvironment of the tumour, some of which are secreted by the neoplastic cells themselves^9^.

We have previously evaluated the presence of M1 and M2 macrophages in a large cohort of CRC patients^1^. We found that CRCs were infiltrated by M1 macrophages, and even though accompanied by M2 macrophages, macrophage infiltration inversely correlated to tumor stage and resulted in an improved patient prognosis in this disease. In this study, we evaluated the distribution and prognostic value of M1 (NOS2^+^) and M2 (CD163^+^) macrophages in a cohort of 234 PC patients. We found a significantly decreased M1/M2 ratio in PC compared to CRC patients, and infiltrating M2 macrophages correlated to a poor patient prognosis. To explore the opposing roles of the immune response in CRC and PC in more detail, we used an *in vitro* cell culture model of tumour-activated macrophages (TaMs). We found that TaMs induced by secreted factors from CRCs or PCs were phenotypically altered and differentially affected lymphocyte activation and polarization, which could be reflected by analysis of the distribution of lymphocyte subsets in CRC and PC tumour tissue. Our findings support the importance of a “good inflammatory response” in the prognosis of CRC patients, and suggest that manipulations of the macrophage phenotype may be important in the development of new treatment strategies in PC and other cancers where immune infiltration is generally linked to poor prognosis. Furthermore, characterization of the immune response could contribute important prognostic information.

## Results

### Macrophages infiltrating PC are mainly of the M2 type resulting in a poor prognosis

**Figure 1:**
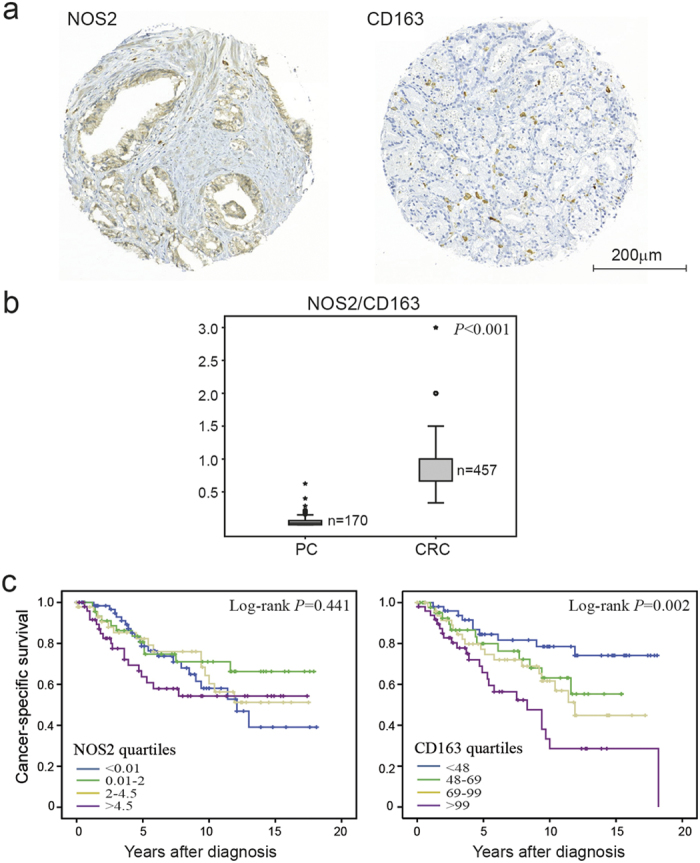
Immunohistological evaluation of M1 and M2 macrophage phenotypes in PC. (**a**) Representative light microscopic images of immunohistochemical staining of NOS2 (M1 marker) and CD163 (M2 marker) in PC. (**b**) The NOS2/CD163 quotient in PC specimens (n = 170) as compared to previously published data in CRC specimens (n = 457)^1^, illustrated by box plots. The levels for PC are presented as a ratio between the individual median number of NOS2- and CD163-positive cells in each tumour specimen, and for CRC as a ratio between NOS2- and CD163-positive cells, scored on a semiquantitative scale. Outlier values (o) and far-out values (*) are indicated. (**c**) Kaplan-Meier plots of cancer-specific survival in PC cases according to levels of NOS2- and CD163-positive cells, presented as quartiles of the individual median numbers.

We have previously evaluated the presence of M1 (NOS2^+^) and M2 (CD163^+^) macrophages by immunohistochemistry in a large cohort (n = 485) of CRC patients^1^. Here, we evaluated the distribution of these M1 and M2 macrophage markers and their impact on patient prognosis in a PC cohort (n = 234). We found that PC generally showed a lower degree of macrophage infiltration than CRC (Fig. 1a and Supplementary Figure S1) with a significantly reduced M1/M2 macrophage ratio (Fig. 1b). The majority of macrophages in PC were of an M2 macrophage phenotype, and a high number of infiltrating M2 macrophages was associated with poor patient prognosis (Fig. 1c). In addition, infiltration of M2 macrophages was associated with a higher Gleason score and increased incidence of metastases at diagnosis (Table 1), but provided independent prognostic information from metastases at diagnosis (HR 1.98, 95% CI 1.17–3.33, *P *= 0.011), but not from Gleason score (HR 0.84, 95% CI 0.49–1.46, *P *= 0.54), in multivariable Cox regression analysis.

### Factors secreted by CRC cells and PC cells induce phenotypic changes in macrophages

**Figure 2:**
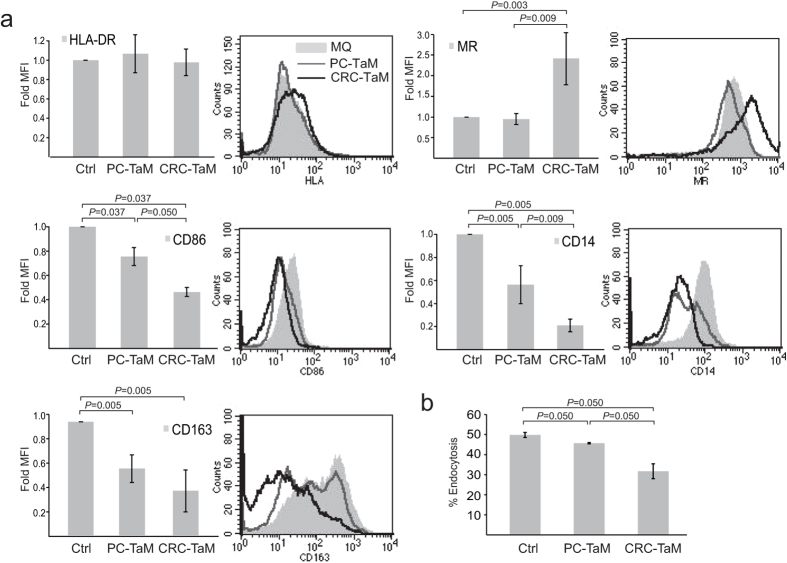
Evaluation of TaM phenotypes. (**a**) Expression of extracellular markers by TaMs. Differentiated macrophages (Ctrl) or PC-TaMs or CRC-TaMs were analysed by immunostaining and flow cytometry. Bars indicate relative mean fluorescense intensity (MFI) ± SD of three or more independent experiments, where each sample was normalized against its respective isotype control. Peaks indicate MFI histograms from one representative experiment. (**b**) The endocytic ability of differentiated macrophages (Ctrl), PC-TaMs and CRC-TaMs was evaluated by measuring endocytic uptake of FITCH-Dextran followed by flow cytometric analysis. Shown is percentage endocytosis ± SD. Significant *P*-values are indicated.

To be able to explore the differences between CRC and PC in more detail, we turned to an *in vitro* cell culture system of tumour activated macrophages (TaMs). TaMs were achieved by differentiating monocytes from healthy donors with macrophage colony stimulating factor (M-CSF) in the presence of conditioned medium from human Sw480 CRC cells (CRC-TaMs) or 22Rv1 PC cells (PC-TaMs). TaM-related markers were analysed by flow cytometry (Fig. 2a). The TaM populations expressed significantly reduced levels of the co-stimulatory molecule CD86 compared to M-CSF-stimulated control macrophages, and the decrease was most evident in CRC-TaMs. Expression of the macrophage marker CD14 and that of the M2 macrophage marker CD163 was also significantly down-regulated in both TaM populations, with the most prominent effect being seen in CRC-TaMs. When comparing to a previously published study, low levels of CD14 and CD163 as seen in CRC-TAMs could be indicative of a more M1-like phenotype^10^. On the other hand, expression of the M2 marker, MR, was significantly upregulated in CRC-TaMs. The endocytic function of the different subsets of TaMs was also investigated in our *in vitro* cell culture system. Cells were differentiated into the different macrophage phenotypes, and monitored by their ability to endocytose fluorescent-labelled dextran. CRC-TaMs had significantly less endocytic function than control macrophages and PC-TaMs (Fig. 2b), which could also indicate a more M1-like phenotype^10^. In conclusion, the macrophage phenotype is changed by tumour secreted factors from both CRC and PC, generally with a stronger effect induced by factors secreted from CRCs.

### Macrophages induced by secreted factors from CRC cells have a proinflammatory cytokine and chemokine expression profile

Using a PCR-based cytokine and chemokine gene expression array, we further analysed the cytokine and chemokine expression of CRC-TaMs and PC-TaMs. We found a changed expression of proinflammatory cytokines and chemokines involved in T cell recruitment and polarization (Table 2). Differentially expressed genes, potentially involved in regulating inflammation and the direction of the T cell response, were verified by RT-PCR (Supplementary Figure S2). The expression of the M1 macrophage proinflammatory cytokine IL6 and the macrophage stimulatory cytokine CSF1 (also denoted M-CSF) was significantly higher in CRC-TaMs. The M2 typical anti-inflammatory cytokine TGFB2 was instead expressed at significantly higher levels in PC-TaMs than in CRC-TaMs. CRC-TaMs further expressed significantly higher levels of chemokines associated with acute inflammation and Th1/CTL responses (i.e. CCL7, CXCL2, CXCL5, and CXCL16). PC-TaMs tended to express higher levels of the chemokine CCL22 supporting Th2/Treg responses and significantly higher levels of CXCL12, a chemokine with multiple functions in cancer, including Treg skewing, immune suppression, stimulation of angiogenesis, and promotion of the migration of cancer cells (PC) to distant metastatic sites (for a review see ref.^11^). Expression of CCL24, which has been implicated in Th2/Treg recruitment, was however increased in CRC-TaMs. These results correlate well to our findings of the distribution of M1 and M2 macrophage subsets in CRC and PC patients.

**Figure 3:**
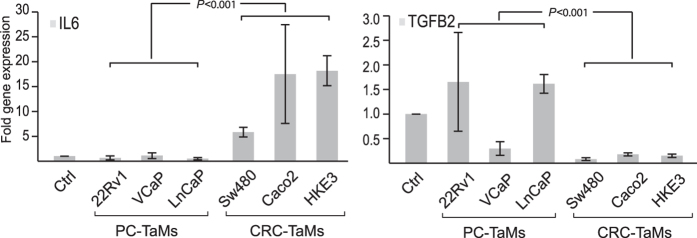
Expression of IL6 and TGFB2 in TaMs induced by conditioned medium from PC cells 22Rv1 (22Rv1-TaM), VCaP (VCaP-TaM) and LnCAP (LnCaP-TaM), or CRC cells Sw480 (Sw480-TaM), Caco2 (Caco2-TaM) and HKE3 (HKE3-TaM). Shown is the fold gene expression from three or more independent experiments ± SD, with Ctrl macrophages set as 1. *P*-values for comparisons between TaMs induced by PC and CRC cell lines are indicated.

The significantly differentially expressed cytokines and chemokines were also examined in TaMs induced by conditioned medium from the PC cell lines VCaP and LnCaP, as well as the CRC cell lines Caco2 and HKE3. IL6 was found to be repeatedly and significantly more highly expressed in TaMs induced by CRC cell lines, while TGFB2 was more highly expressed in TaMs indued by PC cell lines (Fig. 3). TaMs induced by VCaP did not express such high levels of TGFB2, but these were still higher than in TaMs induced by CRC cell lines (*P *= 0.020 for Sw480-TaM; *P *= 0.195 for Caco2-TaM; *P *= 0.028 for HKE3-TaM) (Fig. 3). For the remaining cytokines and chemokines, each cell line induced an individual expression profile and no general pattern was found that clearly distinguished between TaMs induced by conditioned medium from CRC cells and PC cells (Supplementary Figure S3), likely reflecting the heterogeneity of the different cancers.

### CRC-TaMs and PC-TaMs differentially affect the T cell response

**Figure 4:**
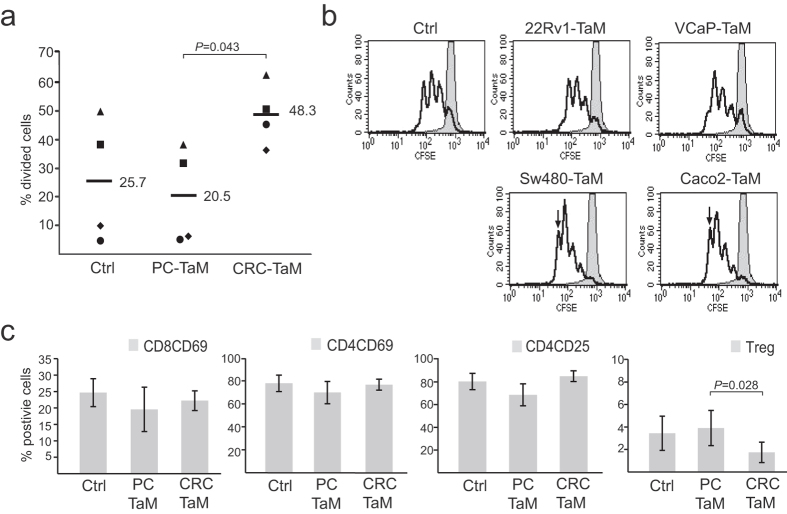
T cell proliferation and activation in response to TaMs. CFSE-labeled T cells were stimulated *in vitro* with anti-CD3 and anti-CD28 plus differentiated macrophages (Ctrl), PC-TaMs or CRC-TaMs for 72 h or left unstimulated. Proliferation of T cells was analysed by flow cytometry. (**a**) Individual percentages of divided cells in the second to fourth cell division. Each dot represents one individual and one separate experiment and horizontal bars indicate mean values. (**b**) Representative CFSE histograms with T cells co-cultured with TaMs induced by conditioned medium from PC cells 22Rv1 (22Rv1-TaM) and VCaP (VCaP-TaM), or CRC cells Sw480 (Sw480-TaM) and Caco2 (Caco2-TaM). The bold line represents T cells stimulated by macrophages and the filled line unstimulated control. Arrow indicates cells in the fourth round of cell division. (**c**) Expression of T cell activation markers CD69 and CD25 after co-culture with PC-TaMs or CRC-TaMs, as detected by immunostaining and flow cytometry. The population of Tregs is defined as CD3^+^CD4^+^CD25^+^CD127^−^ cells. Shown are percentage positive cells ± SD from three independent experiments. Significant *P*-values are indicated.

To determine whether the different cytokine patterns seen in CRC-TaMs and PC-TaMs differentially affected the lymphocyte response, we performed *in vitro* T cell interaction experiments. Autologous T cells were purified and co-cultured with differentiated TaM populations for three days, after which T cell proliferation was analysed by CFSE staining and flow cytometry. Lymphocytes activated in the presence of CRC-TaMs, compared to PC-TaMs, displayed significantly higher proliferation (Fig. 4a). Moreover, we compared TaMs induced by conditioned medium from VCaP-PC and Caco2-CRC cell lines. In lymphocytes activated by TaMs induced by CRC cell lines, more cells were found in the third to fourth round of cell division than in lymphocytes activated by TaMs induced by PC cell lines (Fig. 4b). Furthermore, the activation of T cells was evaluated by flow cytometric analysis using the markers CD69 and CD25. Both CD4^+^ and CD8^+^ T cells co-cultured with PC-TaMs showed a tendency towards lower expression of activation markers than T cells co-cultured with CRC-TaMs (Fig. 4c). In addition, T cells co-cultured with CRC-TaMs showed a significantly reduced Treg population (CD3^+^CD4^+^CD25^+^CD127^−^) compared to T cells stimulated with PC-TaMs (Fig. 4c).

**Figure 5:**
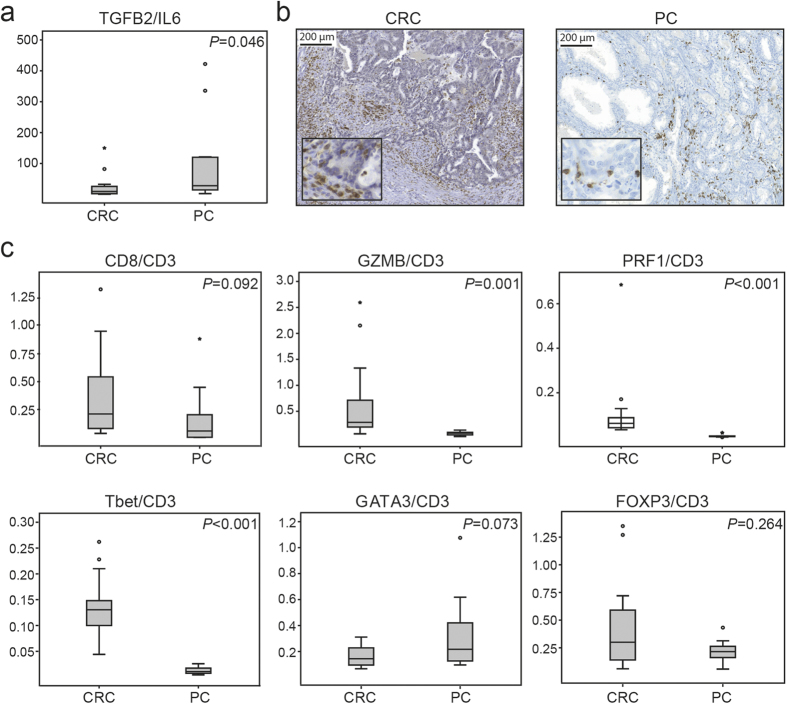
Evaluation of cytokines and T cell phenotypes in CRC specimens (n = 14) and PC specimens (n = 12). (**a**) Analysis of IL6 and TGFB2 expression by RT-PCR. Shown is a box plot of the ratio between TGFB2 and IL6. (**b**) Representative light microscopic images of immunohistochemical staining of the general T cell marker CD3. (**c**) Expression of T cell markers, evaluated by RT-PCR and illustrated with box plots. The levels are represented as a ratio between the mean of 2-∆Ct for each marker in relation to CD3. Outlier values (o) and far-out values (*) are indicated.

To investigate whether our *in vitro* findings would be reflected in tumour specimens from CRC and PC, we evaluated the expression of IL6 and TGFB2 in CRC specimens (n = 14) and PC specimens (n = 12) by RT-PCR. PC specimens had a significantly higher TGFB2/IL6 expression ratio than CRC specimens (Fig. 5a), confirming the results from our *in vitro* studies (Fig. 3). We then evaluated the infiltration of CD3^+^ T lymphocytes in CRC and PC specimens and found that they were both infiltrated by CD3^+^ T lymphocytes (Fig. 5b). We also analysed the presence of different T lymphocyte subtypes in the CRC and PC specimens by comparing the expression of different T cell markers in relation to CD3 expression by RT-PCR (Fig. 5c). There tended to be higher expression of CD8, the marker for CTLs, in CRC specimens. Furthermore, the expression of granzyme B (GZMB) and perforin1 (PRF1), markers of cytolytic activity, were significantly higher in CRC specimens than in PC specimens, where they were almost absent. In addition, expression of the Th1 marker Tbet was significantly higher in CRC specimens than in PC specimens, while GATA3, a marker for Th2 cells, tended to show higher expression in PC tissue. These results suggest that the recruitment and activation of CTLs in CRC is more effective than in PC. FOXP3, a marker for Tregs, was expressed both in CRC and PC specimens but with no significant difference between the cancers.

## Discussion

To gain a better understanding of how CRC and PC differentially affect the immune composition, we compared the distribution and prognostic importance of M1 and M2 macrophages in these cancers. PCs were mainly infiltrated by M2 macrophages and M2 macrophage infiltration was associated with high Gleeson score, increased incidence of metastases and a poor patient prognosis. These findings were in stark contrast to the findings in CRC, where M1 and M2 macrophage infiltration was inversely correlated to tumor stage and strongly linked to an improved prognosis^1^. However, in CRC the ratio of M1/M2 macrophages was much higher than in PC, suggesting that in CRC the function of the M1 macrophages may be dominating.

We next used an *in vitro* cell culture model to study how macrophages respond to secreted factors from CRC or PC cells, and subsequently how these different macrophage subsets affect activation and polarization of lymphocytes. We found that factors secreted by CRC cells triggered a proinflammatory macrophage phenotype, while secreted factors from PC cells induced an immunosuppressive macrophage phenotype. The different macrophage phenotypes were in turn found to affect T cell proliferation and activation in tumour-specific ways. These results could be reflected in tumour specimens, with a more activated T cell profile in CRC and a more immunosuppressive profile in PC. Our results suggest that the opposite effects on the macrophage phenotype by tumour secreted factors, may contribute to the opposing role of immune infiltration that we see in CRC and PC.

It has been shown repeatedly that macrophage infiltration is a good prognostic indicator in CRC^2^,^12^,^13^,^14^. In PC, the importance of macrophage infiltration is not as thoroughly studied, but we and others have shown that macrophage infiltration is a poor prognostic factor^3^,^15^,^16^,^17^. In addition, we have shown that extratumoural macrophages promote tumour and vascular growth in an *in vivo* prostate tumour model^18^. However, there have been results published that contradict this^19^, underscoring the need for further evaluation and standardization of quantification protocols. Only a few studies have compared the different contributions of M1 and M2 macrophages in tumour progression and patient prognosis. We have previously shown that the presence of M1 macrophages gives a better prognosis in CRC patients, irrespective of the accompanying infiltration by M2 macrophages^1^. Here, we evaluated the distribution of M1 (NOS2^+^) and M2 (CD163^+^) macrophages in a cohort of PC patients. PCs showed a much lower M1/M2 ratio than CRCs and M2 macrophage infiltration was correlated with tumour progression and poor prognosis. Our results are supported by a study by Ong *et al.* in which TaMs in CRC were found to be linked to a proinflammatory phenotype^20^. The authors also showed, as in our study, that the TaMs induced by CRC cells could support a Th1 response. That particular study used spheroids, which allowed cellular contacts between tumour cells and macrophages, and might implicate a risk of self-nonself recognition. In the present study we found that tumour-secreted factors alone were sufficient. Moreover, Algars *et al.* demonstrated in their study that a lower M1/M2 macrophage ratio in CRC resulted in more recurrent disease^12^. A high M1/M2 ratio has also been correlated to improved survival in NSCLC^21^, suggesting that the presence of M1 macrophage infiltration in general is a good prognostic marker. Lanciotti *et al.* attempted to evaluate both M1 and M2 macrophages in PC, but the M1 macrophages were singled out from expression of the general macrophage marker CD68, a marker that has also been shown to be expressed by CD163 positive cells^1^. However, infiltration of M2 (CD163^+^) macrophages was correlated to disease progression and high total macrophage infiltration was correlated to poor patient survival^16^. It should be noted that the classical view of macrophage phenotypes separated into distinct M1 and M2 phenotypes is likely an oversimplification. In reality, macrophages are very plastic cells and can be redirected *in vitro* towards the opposite functional phenotype^10^,^22^. Further characterization and improved markers are required for more comprehensive analyses of the macrophage phenotypes and their roles in tumour progression.

Using an *in vitro* cell culture, we found that TaMs induced by secreted factors from CRCs or PCs were phenotypically altered. The most solid finding, reproduced using several different CRC and PC cell lines, was that the cytokine IL6 was found to be significantly more highly expressed in TaMs induced by conditioned medium from CRC cell lines, while TGFB2 was more highly expressed in TaMs induced by conditioned medium from PC cell lines. These differences could also be reflected in tumour specimens. The complete actions of cytokines and chemokines are generally complex, with functions in tumour rejection or tumour promotion depending on the context of the tumour microenvironment. Inflammatory conditions have been known to drive tumour formation and growth of CRC through production of cytokines such as IL6^23^. However, IL6 also acts as an important driver of acquired immunity^24^. TGFB is an important suppressor of inflammation and immunity, but at the same time it also has multiple roles in tumour invasion and metastasis^25^. General chemokine differences related to cancer origin were not detected. However, the effect of TaMs on lymphocyte proliferation was consistent within CRC and PC cell lines, suggesting that the overall response may be similar. These results indicate that phenotypic variations in macrophages are likely due to both tumour- and organ-specific differences.

To study how the different TaM phenotypes that appear in CRC and PC affect the other arms of the immune system, we investigated their ability to activate lymphocytes using *in vitro* co-culture experiments. We found that TaMs induced by conditioned medium from CRC cell lines stimulated T cell proliferation and decreased the number of Tregs, when compared to TaMs induced by conditioned medium from PC cells. Analysis of T cell subtypes in tumour tissue showed increased amounts of Th1 cells and active CTLs in CRC compared to PC. Interestingly, Th1 cells have been shown to be able to support an M2-to-M1 transition^26^. In PC, the Th2 lymphocytes were predominant instead and the CD3^+^ lymphocytes were mainly negative for perforin and granzyme B suggesting that they were functionally inactive. These results are consistent with the data of Ebelt *et al.* who showed that infiltrating CD8^+^ T cells were rare and quiescent in the PC microenvironment^27^. Tregs were identified in both CRC and PC specimens, with no significant differences. It might be that in CRC, Tregs are present as a consequence of immune activity in order to normalize the activity of inflammatory factors that might otherwise drive tumour progression, and to prevent excessive reactions. In support of this theory, infiltrating Tregs in CRC have been associated with an improved survival^28^,^29^,^30^,^31^. On the contrary, in PC an increased number of Tregs have been shown to be associated with an adverse clinical outcome^32^,^33^. Several studies have tried to describe infiltration of different T cell subtypes and their prognostic importance in cancer. According to a recent meta-analysis Th1/CTLs are associated with prolonged survival in CRC and many other cancers, while a Th2/Treg profile is generally associated with a poor prognosis or has no impact^5^. In PC, the prognostic importance of lymphocyte infiltration is still being debated, but several studies have suggested that immune infiltration is dominated by a Th2/Treg profile and is a predictor of poor prognosis^32^,^33^,^34^. Work to further characterise the immune populations in these different cancers and their prognostic relation is of urgent need.

One explanation for the opposing roles of the immune response in CRC and PC could lie in the fundamental differences between these organs. The intestine is an organ that is subjected to many immune challenges, which has led to a number of important adaptations to maintain local tissue homeostasis^35^,^36^. The prostate gland, is a more sterile environment and has a strong immunoregulatory capacity^37^. An interesting hypothesis is that viral or bacterial antigens may trigger active immune components in CRC. Here we designated immune activating functions in CRC to factors secreted by the tumour epithelial cells themselves without such influences and we have initiated the search for tumour secreted factors with a potential immune activating role.

In conclusion, CRCs are infiltrated by M1 macrophages which contribute to an improved patient prognosis. PCs, on the contrary, are sparsely infiltrated by M1 macrophages, and M2 macrophage infiltration contributes to a poor prognosis. The opposing prognostic roles of macrophage infiltration in these cancers can partly be explained by that tumour secreted factors can drive the macrophage phenotype in different directions, which in turn will have effects on the overall organization of the immune response. By comparing CRC and PC, we have taken important steps towards understanding the biological background of the opposing roles of immune infiltration in different cancers, which may lead to the development of important new treatment strategies.

## Materials and Methods

### Patient samples

The handling of tissue samples and patient data in the present study was approved by the research ethical committee at Umeå university hospital (Regional Ethical Review Board), Umeå, Sweden. Tissue samples were registered as a case number and year in a database used for the analyses, with no names or personal identification number indicated. All methods were carried out in accordance with the approved guidelines.

Expression of NOS2 and CD163 was evaluated in 234 tissue specimens collected from patients with voiding problems who underwent transurethral resection of the prostate (TURP) at the hospital in Västerås, Sweden, between 1975 and 1991 (prior to the PSA era). The material was collected according to Swedish regulations at a time when informed consent was not required. The research ethical committee at Umeå university hospital approved of the study and waived the need for consent. Histological evaluation was used to identify PCs. The patients had not received anti-cancer therapy before TURP. Radionuclide bone scan was used to detect bone-metastases and the tumors were Gleason-graded by a single pathologist. After diagnosis the patients were, according to the therapy traditions in Sweden at that time, managed by watchful waiting. Symptomatic treatment (androgen ablation or radiation treatment) was introduced in patients who developed symptoms from metastases. From the formalin fixed paraffiin embedded tissue samples, tissue micro arrays (TMAs) were constructed using a Beecher Instrument (Sun Prairie, WI, USA). The TMAs contained 5–8 samples of tumor tissue and 4 samples of non-malignant tissue from each patient. For more details on this patient cohort and the TMAs we refer to previously published information^38^,^39^. The numbers of NOS2 positive cells were counted on the total surface of the normal and tumor tissue cores. Due to high abundance, CD163 positive cells were counted in the lower, right quartile of each core and then multiplied by four to get an approximate number of the total amount. Analysis of median, mean and maximum patient TMA levels gave similar results (results not shown), but median values were used in correlation and survival analyses.

For *in situ* evaluation of T cells in tumour sections, tissue samples from 14 patients surgically resected for CRC and 12 PC patients treated with radical prostatectomy were collected at the Department of Surgery, Umeå University Hospital, Umeå, Sweden. Formalin fixed paraffin embedded (FFPE) CRC tissues were from patients surgically resected for CRC between 1995 and 2003. The patients verbally gave their informed consent. This consent was documented in each patient record, and considered by the ethical committee to be sufficient. From all patients, clinicopathologic and molecular variables have been well defined according to procedures described in Dahlin *et al.*^40^. Median age for the patients was 75 years (range 43–88 years). One tumor was classified as stage I, seven as stage II, four as stage III and two as stage IV. Two tumors were microsatellite instable (MSI) and twelve were microsatellite stable (MSS). Prostate cancer tissue was obtained from patients treated with radical prostatectomy between 2008 and 2009. The patients gave written informed consent and the study was approved by the research ethical committee. Frozen samples and FFPE whole mount prostate tissue were available from each patient, according to a tissue handling procedure previously described in Hörnberg *et al.*^41^. Median age for the patients was 61 years (range 48–68 years) and median PSA were 12 ng/ml (range 3.5–24 ng/ml). Ten of the patients had tumors graded as Gleason score (GS) 7 and two as GS 8. Three of the tumors were in pathological stage T2 and nine in stage T3.

### Immunohistochemistry of tumour tissue specimens

For immunohistochemical staining, 4-μm formalin fixed and paraffin embedded (FFPE) sections were cut, dried, de-waxed, and rehydrated. CD3 (LN10; dilution 1:50; Novocastra), CD163 (NCL-CD163; dilution 1:100; Novacastra,), and NOS2 (ab15323, dilution 1:50; Abcam,) were used on an automated Ventana Benchmark Ultra staining machine using the iVIEW DAB Detection Kit for visualization (Ventana,). The slides were counterstained with hematoxylin and scanned using the Panoramic 250 FLASH scanner (3D Histech) and visualized with the Panoramic viewer software (3D Histech).

### *In vitro* cell culture experiments

The CRC cell lines SW480, Caco2, and HKE3 and the PC cell lines 22Rv1, VCaP, and LNCaP (all from ATCC, verified using short tandem repeat profiling by Biosynthesis) were grown in RPMI 1640 or DMEM GluTaMax^TM^ (Invitrogen) supplemented with 10% fetal bovine serum (FBS) (Life technologies), 1 mM sodium pyruvate (Invitrogen), penicillin (100 U/ml; Invitrogen) and streptomycin (10 mg/ml; Invitrogen) at 37 °C with 5% carbon dioxide. CRC and PC cells were grown to approximately 90% confluence for 48 hours, and conditioned medium was collected, centrifuged at 3,000 rpm for 30 minutes and stored at −80 °C until use.

Human monocytes were obtained from whole blood from anonymous healthy donors, or from blood donor buffy coats (according to local guidelines at the Blood center, Umeå University Hospital). Peripheral blood mononuclear cells (PBMCs) were purified from blood donor buffy coats by dextran sedimentation and Fiqoll-Paque gradient centrifugation. Monocytes were further purified from the PBMCs by magnetic-activated cell sorting (MACS) using the Monocyte Isolation Kit II (Miltenyi Biotec), resulting in a monocyte population of >95% purity. TaMs were achieved by incubating monocytes in tumour conditioned medium (or RPMI with 10% FBS as control) supplemented with 50 ng/ml M-CSF (216-MC-025; R&D Systems) and antibiotics. The medium was changed at day 3, and at day 6 the cells were harvested and subjected to further analysis. For autologous interaction studies, PBMCs were isolated from whole blood by gradient centrifugation on Lymphoprep (Nycomed) and monocytes were further purified by MACS, stimulated and evaluated as above. At day 6, T cells were purified by Pan T Cell Isolation Kit II (Milteneyi Biotec) from PBMCs isolated from whole blood from the same individual. The purity of sorted T cells was >95%. T cells were co-cultured with autologous macrophages differentiated in the presence of conditioned medium from tumour cells (or RPMI containing 10% FBS as control) in a T cell to macrophage ratio of 4:1. The cells were incubated for 72 h in flat-bottomed 96-well plates previously coated with anti-CD3 (1 μg/ml clone HIT3a; BD PharMingen) and anti-CD28 (1 μg/ml clone 37.51; BD PharMingen) before analysis.

### Expression of macrophage and lymphocyte markers

Flow cytometry was used to analyse expression of macrophage and lymphocyte specific cell surface markers (BD FACSCalibur^TM^ Flow Cytometer) using phycoerythrin (PE)-conjugated monoclonal antibody against CD14 (clone M5E2, BD Pharmingen), CD1a (clone HI149, ImmunoTools) and CD4 (RPAT4, BD Biosciences); PE-Cyanin5 (Cy5)-conjugated monoclonal antibody against CD25 (MA251, BD Biosciences) and CD69 (FN50, BD Biosciences); Fluorescein isothiocyanate (FITC)-conjugated monoclonal antibody against CD86 (clone FUN-1, BD Biosciences), HLA-DR (clone MEM12, Abcam) and CD3 (UCHT1, R&D Systems); Allophycocyanin (APC)-conjugated monoclonal antibody against Mannose receptor (MR) (clone 15-2, Biolegend), CD163 (clone 215927, R&D Systems) and CD8 (clone RPA-T8, BD Biosciences); and AlexaFluor 647- conjugated monoclonal antibody against CD127 (HIL-7R-M21, BD Biosciences). Before staining, Fc receptors were blocked with 5% human TruStain FCX^TM^ (Biolegend) for 10 minutes. For all experiments, matched isotype controls were included. Data were analysed with CellQuest Pro software (Tree Star) after gating on the macrophage or lymphocyte population in the FSC/SSC window. Data from 10–20 000 gated events was collected.

### Endocytosis

To monitor endocytic function of the different macrophage populations, FITC-dextran internalization was measured. TaMs were harvested, the cell numbers were adjusted to 5 × 10^5^ cells in 1 ml of RPMI medium, and they were seeded in a 24-well plate. They were pre-incubated on ice for 30 minutes and then incubated with 20 μg/ml FITC-dextran (Sigma-Aldrich) for 15 min at 37 °C or 4 °C, to detect cell surface binding. The cells were washed three times using 1 ml of cold PBS containing 5% FBS and percentage endocytosis was determined by flow cytometry.

### Cell proliferation

T cell proliferation was measured by flow cytometry using carboxyfluorescein diacetate succinimidyl ester (CFSE) (CellTrace^TM^ CFSE Cell Proliferation Kit; Invitrogen). Briefly, 2 × 10^6^ cells were incubated with 0.68 μM CFSE at 37 °C for 10 min, washed with ice-cold culture medium (containing 50% FBS), and then added to the culture at 1 × 10^5^ T cells/well (T cell/macrophage ratio: 4:1) in a 96-well plate. 72 hours later, dividing cells were detected by flow cytometry and analyzed using CellQuest software (BD).

### Analysis of cytokine and chemokine expression

The gene expression profiles of the different macrophage populations were studied with the Cytokines & Chemokines PCR Array (PAHS-150A; SABiosciences) according to the manufacturer’s protocol. Briefly, total RNA was isolated from different cell populations using the RNeasy Plus Mini Kit (Qiagen). cDNA was synthesized using the RT^2^ First Strand Kit. Amplification data (fold changes in C_t_ values of all the genes) were analysed with SABiosciences software. For verification of cytokine and chemokine expression in the different macrophage populations, total RNA was isolated using the RNeasy Plus Mini Kit (Qiagen) and cDNA was synthesized using the Superscript II Reverse Transcriptase (Invitrogen). Primers used for normalization were GAPDH forward: 5′-TGCACCACCAACTGCTTAGC-3′ and reverse: 5′-GGCATGGACTGTGGTCATGAG-3′ (DNA Technology A/S). For the other genes Quantitect Primer Assays (Quiagen) were used.

For analysis of expression of cytokine genes or lymphocyte markers in CRC tumour tissue, RNA was extracted from 4 FFPE-embedded tumour sections (4 μM) using the High Pure RNA Micro Kit (Roche). For expression analysis in PC tissue, RNA was extracted from 10 cryo-sections using the TRIzol method (Life Technologies). cDNA was prepared using the Superscript VILO cDNA Synthesis Kit (Invitrogen). Primers used were for normalization RPL13A forward: 5′-GTA CGC TGT GAA GGC-3′ and reverse: 5′-GTT GGT GTT CAT CCG-3′. For the other genes Quantitect Primer Assays (Quiagen) were used.

The RT-PCR-reactions were run on a Taqman 7900HT (Applied Biosystems, Life Technologies) and following cycling parameters were used: 50 °C for 2 min and then an initial denaturation at 95 °C for 10 min, followed by 40 cycles of 95 °C for 15 s and 60 °C for 60 s.

### Statistics

Statistical analysis was performed using PASW Statistics 22 (SPSS Inc., Chicago, IL, USA). Correlations between continuous variables were analysed using the Spearman rank correlation test. The non-parametric Kruskal-Wallis *H* and Mann-Whitney *U* tests were used to compare differences in continuous variables between groups. Kaplan-Meier survival analysis was used to estimate cancer-specific survival, and duration of cancer-specific survival was defined as the time from TURP until the date of death from PC. The log-rank test was used to compare differences in outcome between groups. Multivariate survival analyses were performed by using Cox proportional hazard models. All statistical analyses were two-sided and *P*-values ≤0.05 were considered statistically significant.

## Additional Information

**How to cite this article**: Lundholm, M. *et al.* Secreted Factors from Colorectal and Prostate Cancer Cells Skew the Immune Response in Opposite Directions. *Sci. Rep.* **5**, 15651; doi: 10.1038/srep15651 (2015).

## Supplementary Material

Supplementary Information

## Figures and Tables

**Table 1:** Number of NOS2 and CD163 positive cells in relation to clinicopathologic characteristics in PC.

	NOS2	CD163
Frequency	3.4 (±4.6)	77.1 (±38.4)
*n*	208	196
Gleason score		
4–5 (*n *= 46,38)	2.9 (±4.7)	60.1 (±28.5)
6 (*n *= 56,51)	2.6 (±3.3)	63.7 (±31.5)
7 (*n *= 42,45)	3.9 (±3.9)	75.2 (±27.5)
8–10 (*n *= 64,62)	4.0 (±5.8)	100.0 (± 44.8)
*P-value*	0.072	<0.001
Metastases		
No (*n *= 184,178)	3.3 (±4.8)	74.8 (±36.3)
Yes (*n *= 24,18)	3.5 (±2.9)	99.9 (±51.0)
*P-value*	0.173	0.042
CD163		
*n*	170	−
*r*_*s*_	0.286*	−
*P-value*	<0.001*	−

Frequency was presented as mean number of cells ± SD. Kruskal-Wallis or Mann-Whitney tests were used for categorical variables.

^*^Spearman’s rank correlation test was used for continuous variables. *Abbreviations: r*_*s*_, Spearman’s rank correlation coefficient; *n*, number of patients.

**Table 2:** Differentially expressed genes in PC-TaMs or CRC-TaMs compared to control macrophages.

Gene symbol	Fold-change	
	PC-TaM	CRC-TaM
*CCL1*†	7.5	46.7
CCL3*	4.5	2.0
CCL5*	2.9	4.1
*CCL7*	3.9	2050.0
*CCL13*	1.2	3610.4
*CCL18*†	2.7	90.5
*CCL22*†	7.5	2.7
*CCL24*†	0.9	1134.9
*CSF1*	2.9	26.5
*CXCL1*	2.5	4.8
*CXCL2*	3.8	10.3
*CXCL5*	11.5	5461.8
*CXCL12*	5.2	−10.0
CXCL16*	3.1	5.5
IL1A*	−4.5	1.2
IL1B*	1.2	7.1
*IL1RN*†	1.9	31.8
IL6*	−4.0	6.8
*IL8*	14.0	73.8
*IL15*	0.9	2.7
IL23A*	14.0	73.8
*LTB*	0.9	7.4
*TGFB2*†	0.8	−8.3
*TNFSF10*	1.1	5.5
*VEGFA*	1.6	4.1

Genes ≥2.5-fold up- or down-regulated with at least one Ct value below 30 are shown. Factors defining distinct M1 macrophages

^(*)^ and M2 macrophages

^(†)^ are indicated^7^. See Supplementary Table S1 for complete data set.

## References

[b1] Edin S. *et al.* The distribution of macrophages with a m1 or m2 phenotype in relation to prognosis and the molecular characteristics of colorectal cancer. PLoS One 7, e47045 (2012). 23077543 10.1371/journal.pone.0047045 PMC3471949

[b2] Forssell J. *et al.* High macrophage infiltration along the tumor front correlates with improved survival in colon cancer. Clin Cancer Res 13, 1472–1479 (2007). 17332291 10.1158/1078-0432.CCR-06-2073

[b3] Lissbrant I. F. *et al.* Tumor associated macrophages in human prostate cancer: relation to clinicopathological variables and survival. Int J Oncol 17, 445–451 (2000). 10938382 10.3892/ijo.17.3.445

[b4] Zhang Q. W. *et al.* Prognostic significance of tumor-associated macrophages in solid tumor: a meta-analysis of the literature. PLoS One 7, e50946 (2012). 23284651 10.1371/journal.pone.0050946 PMC3532403

[b5] Fridman W. H., Pages F., Sautes-Fridman C. & Galon J. The immune contexture in human tumours: impact on clinical outcome. Nat Rev Cancer 12, 298–306 (2012). 22419253 10.1038/nrc3245

[b6] Biswas S. K. & Mantovani A. Macrophage plasticity and interaction with lymphocyte subsets: cancer as a paradigm. Nat Immunol 11, 889–896 (2010). 20856220 10.1038/ni.1937

[b7] Mantovani A. *et al.* The chemokine system in diverse forms of macrophage activation and polarization. Trends Immunol 25, 677–686 (2004). 15530839 10.1016/j.it.2004.09.015

[b8] Mosser D. M. & Edwards J. P. Exploring the full spectrum of macrophage activation. Nat Rev Immunol 8, 958–969 (2008). 19029990 10.1038/nri2448 PMC2724991

[b9] Ruffell B., Affara N. I. & Coussens L. M. Differential macrophage programming in the tumor microenvironment. Trends Immunol 33, 119–126 (2012). 22277903 10.1016/j.it.2011.12.001 PMC3294003

[b10] Edin S., Wikberg M. L., Rutegard J., Oldenborg P. A. & Palmqvist R. Phenotypic skewing of macrophages *in vitro* by secreted factors from colorectal cancer cells. PLoS One 8, e74982 (2013). 24058644 10.1371/journal.pone.0074982 PMC3776729

[b11] Karin N. The multiple faces of CXCL12 (SDF-1alpha) in the regulation of immunity during health and disease. J Leukoc Biol 88, 463–473 (2010). 20501749 10.1189/jlb.0909602

[b12] Algars A. *et al.* Type and location of tumor-infiltrating macrophages and lymphatic vessels predict survival of colorectal cancer patients. Int J Cancer 131, 864–873 (2012). 21952788 10.1002/ijc.26457

[b13] Lackner C. *et al.* Prognostic relevance of tumour-associated macrophages and von Willebrand factor-positive microvessels in colorectal cancer. Virchows Arch 445, 160–167 (2004). 15232739 10.1007/s00428-004-1051-z

[b14] Zhou Q. *et al.* The density of macrophages in the invasive front is inversely correlated to liver metastasis in colon cancer. J Transl Med 8, 13 (2010). 20141634 10.1186/1479-5876-8-13 PMC2841127

[b15] Nonomura N. *et al.* Infiltration of tumour-associated macrophages in prostate biopsy specimens is predictive of disease progression after hormonal therapy for prostate cancer. BJU international 107, 1918–1922 (2011). 21044246 10.1111/j.1464-410X.2010.09804.x

[b16] Lanciotti M. *et al.* The role of M1 and M2 macrophages in prostate cancer in relation to extracapsular tumor extension and biochemical recurrence after radical prostatectomy. BioMed research international 2014, 486798 (2014). 24738060 10.1155/2014/486798 PMC3967497

[b17] Tidehag V. *et al.* High density of S100A9 positive inflammatory cells in prostate cancer stroma is associated with poor outcome. Eur J Cancer 50, 1829–1835 (2014). 24726733 10.1016/j.ejca.2014.03.278

[b18] Halin S., Rudolfsson S. H., Van Rooijen N. & Bergh A. Extratumoral macrophages promote tumor and vascular growth in an orthotopic rat prostate tumor model. Neoplasia 11, 177–186 (2009). 19177202 10.1593/neo.81338 PMC2631142

[b19] Shimura S. *et al.* Reduced infiltration of tumor-associated macrophages in human prostate cancer: association with cancer progression. Cancer Res 60, 5857–5861 (2000). 11059783

[b20] Ong S. M. *et al.* Macrophages in human colorectal cancer are pro-inflammatory and prime T cells towards an anti-tumour type-1 inflammatory response. European journal of immunology 42, 89–100 (2012). 22009685 10.1002/eji.201141825

[b21] Ohri C. M., Shikotra A., Green R. H., Waller D. A. & Bradding P. Macrophages within NSCLC tumour islets are predominantly of a cytotoxic M1 phenotype associated with extended survival. Eur Respir J 33, 118–126 (2009). 19118225 10.1183/09031936.00065708

[b22] Stout R. D. *et al.* Macrophages sequentially change their functional phenotype in response to changes in microenvironmental influences. J Immunol 175, 342–349 (2005). 15972667 10.4049/jimmunol.175.1.342

[b23] Rizzo A., Pallone F., Monteleone G. & Fantini M. C. Intestinal inflammation and colorectal cancer: a double-edged sword? World J Gastroenterol 17, 3092–3100 (2011). 21912451 10.3748/wjg.v17.i26.3092 PMC3158408

[b24] Jones S. A. Directing transition from innate to acquired immunity: defining a role for IL-6. J Immunol 175, 3463–3468 (2005). 16148087 10.4049/jimmunol.175.6.3463

[b25] Ikushima H. & Miyazono K. TGFbeta signalling: a complex web in cancer progression. Nat Rev Cancer 10, 415–424 (2010). 20495575 10.1038/nrc2853

[b26] Heusinkveld M. *et al.* M2 macrophages induced by prostaglandin E2 and IL-6 from cervical carcinoma are switched to activated M1 macrophages by CD4^+^ Th1 cells. J Immunol 187, 1157–1165 (2011). 21709158 10.4049/jimmunol.1100889

[b27] Ebelt K. *et al.* Dominance of CD4^+^ lymphocytic infiltrates with disturbed effector cell characteristics in the tumor microenvironment of prostate carcinoma. The Prostate 68, 1–10 (2008). 17948280 10.1002/pros.20661

[b28] Frey D. M. *et al.* High frequency of tumor-infiltrating FOXP3(+) regulatory T cells predicts improved survival in mismatch repair-proficient colorectal cancer patients. Int J Cancer 126, 2635–2643 (2010). 19856313 10.1002/ijc.24989

[b29] Ladoire S., Martin F. & Ghiringhelli F. Prognostic role of FOXP3+ regulatory T cells infiltrating human carcinomas: the paradox of colorectal cancer. Cancer immunology, immunotherapy: CII 60, 909–918 (2011). 21644034 10.1007/s00262-011-1046-y PMC11028605

[b30] Ling A., Edin S., Wikberg M. L., Oberg A. & Palmqvist R. The intratumoural subsite and relation of CD8 and FOXP3 T lymphocytes in colorectal cancer provide important prognostic clues. Br J Cancer 110, 2551–2559 (2014). 24675384 10.1038/bjc.2014.161 PMC4021513

[b31] Salama P. *et al.* Tumor-infiltrating FOXP3+ T regulatory cells show strong prognostic significance in colorectal cancer. J Clin Oncol 27, 186–192 (2009). 19064967 10.1200/JCO.2008.18.7229

[b32] Davidsson S. *et al.* CD4 helper T cells, CD8 cytotoxic T cells, and FOXP3(+) regulatory T cells with respect to lethal prostate cancer. Mod Pathol 26, 448–455 (2013). 23041830 10.1038/modpathol.2012.164

[b33] Flammiger A. *et al.* High tissue density of FOXP3+ T cells is associated with clinical outcome in prostate cancer. Eur J Cancer 49, 1273–1279 (2013). 23266046 10.1016/j.ejca.2012.11.035

[b34] Sfanos K. S. *et al.* Phenotypic analysis of prostate-infiltrating lymphocytes reveals TH17 and Treg skewing. Clin Cancer Res 14, 3254–3261 (2008). 18519750 10.1158/1078-0432.CCR-07-5164 PMC3082357

[b35] Hooper L. V. & Macpherson A. J. Immune adaptations that maintain homeostasis with the intestinal microbiota. Nat Rev Immunol 10, 159–169 (2010). 20182457 10.1038/nri2710

[b36] Weber B., Saurer L. & Mueller C. Intestinal macrophages: differentiation and involvement in intestinal immunopathologies. Semin Immunopathol 31, 171–184 (2009). 19533135 10.1007/s00281-009-0156-5

[b37] Miller A. M. & Pisa P. Tumor escape mechanisms in prostate cancer. Cancer immunology, immunotherapy: CII 56, 81–87 (2007). 16362411 10.1007/s00262-005-0110-x PMC11041923

[b38] Hammarsten P., Rudolfsson S. H., Henriksson R., Wikstrom P. & Bergh A. Inhibition of the epidermal growth factor receptor enhances castration-induced prostate involution and reduces testosterone-stimulated prostate growth in adult rats. The Prostate 67, 573–581 (2007). 17252557 10.1002/pros.20529

[b39] Egevad L., Granfors T., Karlberg L., Bergh A. & Stattin P. Prognostic value of the Gleason score in prostate cancer. BJU international 89, 538–542 (2002). 11942960 10.1046/j.1464-410x.2002.02669.x

[b40] Dahlin A. M. *et al.* Colorectal cancer prognosis depends on T-cell infiltration and molecular characteristics of the tumor. Mod Pathol 24, 671–682 (2011). 21240258 10.1038/modpathol.2010.234

[b41] Hornberg E. *et al.* Expression of androgen receptor splice variants in prostate cancer bone metastases is associated with castration-resistance and short survival. PLoS One 6, e19059 (2011). 21552559 10.1371/journal.pone.0019059 PMC3084247

