# Supplementary material for: Secreted Factors from Colorectal and Prostate Cancer Cells Skew the Immune Response in Opposite Directions
Source: Sci Rep. 2015 Oct 27;5:15651. doi: 10.1038/srep15651 (PMC4621525; doi:10.1038/srep15651)
Supplement: Supplementary Information [file srep15651-s1.pdf]

Supplementary Information

"Secreted Factors from Colorectal and Prostate Cancer Cells Skew the Immune Response in Opposite Directions"

Marie Lundholm[1], Christina Hägglöf[1], Maria L. Wikberg[1], Pär Stattin[2], Lars Egevad[3], Anders Bergh[1], Pernilla Wikström[1], Richard Palmqvist[1], and Sofia Edin[1,*]

[1]Department of Medical Biosciences, Pathology, Umeå University, Umeå, Sweden.
[2]Department of Surgical and Perioperative Sciences, Urology and Andrology, Umeå University, Umeå, Sweden.
[3]Department of Oncology-Pathology, Karolinska Institute, Stockholm, Sweden.
*sofia.edin@umu.se

Supplementary Figure S1

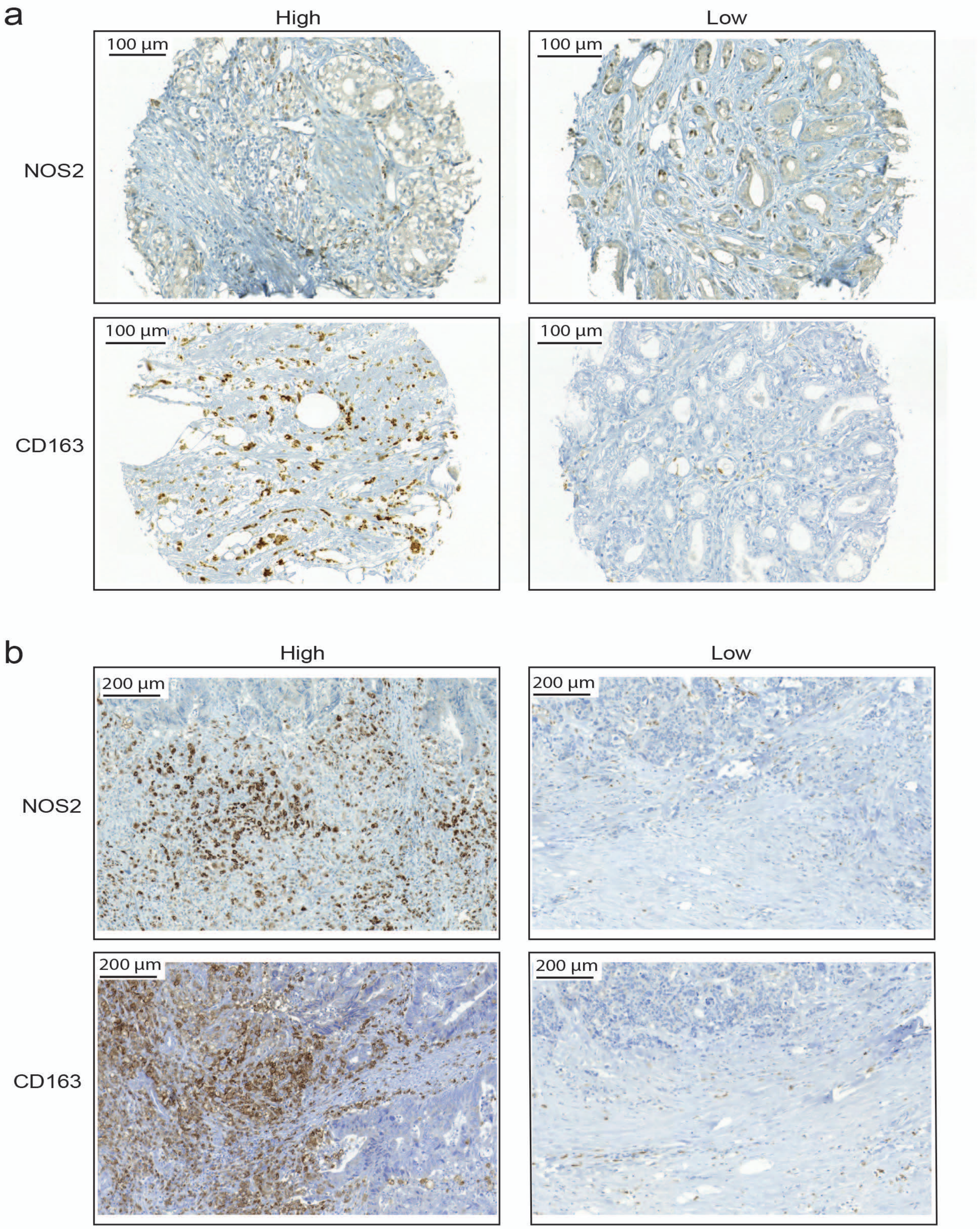

Supplementary Figure S2

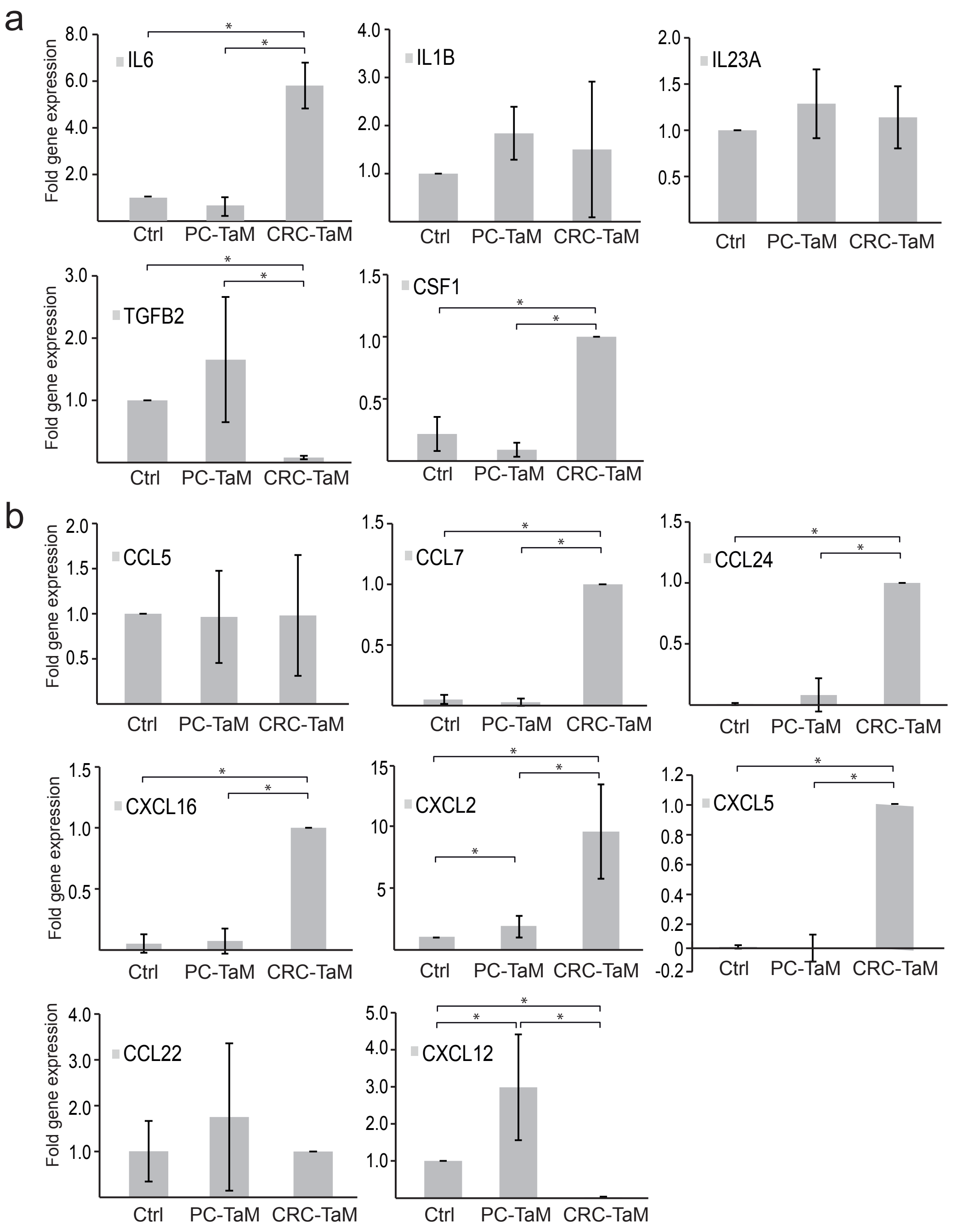

Supplementary Figure S3

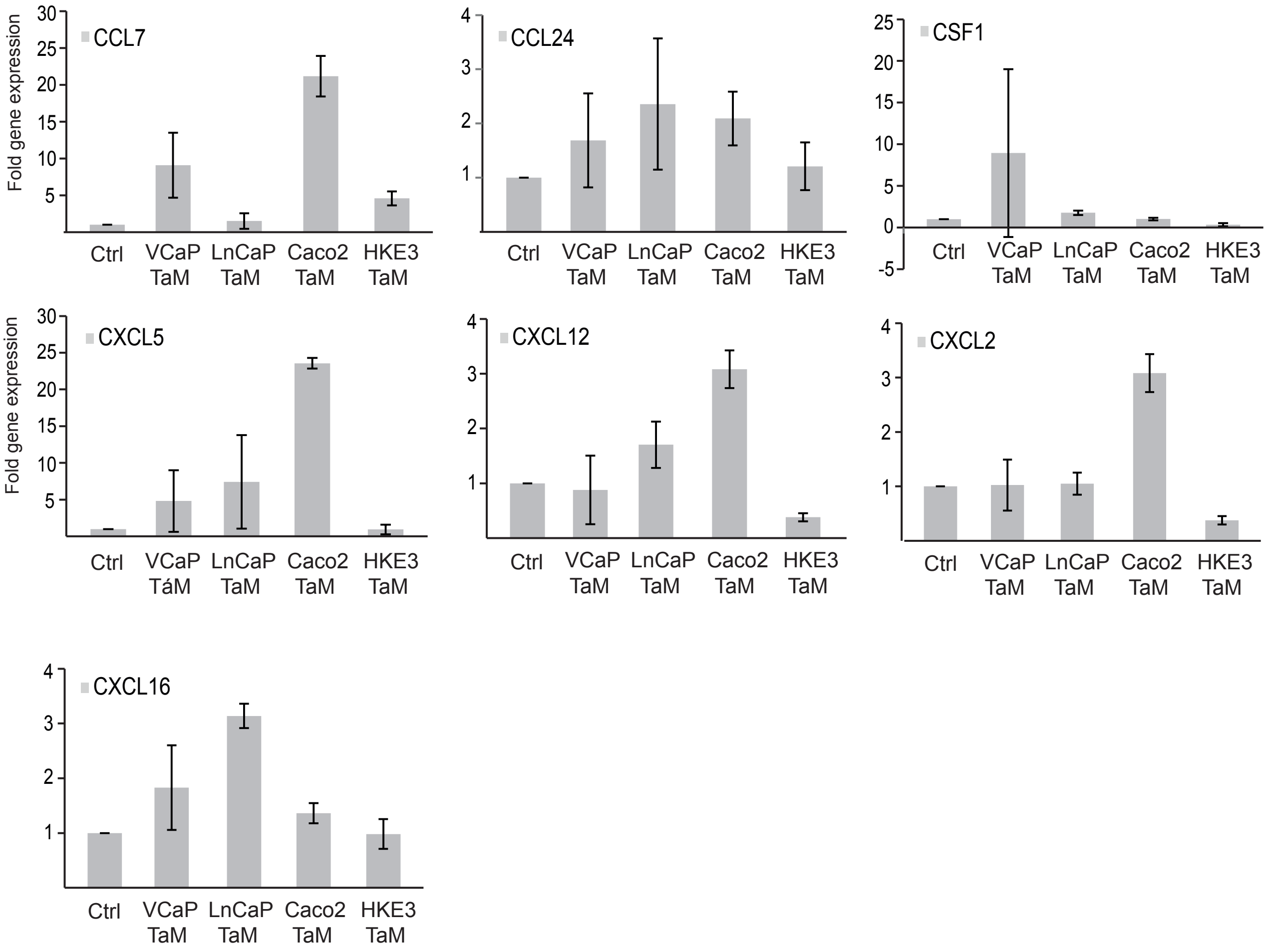

Supplementary Figure Legends

Figure S1. Representative images of immunohistological staining of NOS2 and CD163. Shown are tumor specimens with high and low infiltration of immunoreactive macrophages in PC (a) and CRC (b).

Figure S2. Expression of cytokine and chemokine genes by TaMs. Gene expression of differentiated macrophages (Ctrl) or PC-TaMs or CRC-TaMs was anlayzed by RT-PCR. Shown is the fold gene expression from three or more independent experiments ± SD, with Ctrl macrophages or CRC-TaMs set as 1. Significant differences are indicated by * ($P \leq 0.05$). (A) Expression of proinflammatory and immunosupressive cytokines. (B) Expression of chemokines associated with Th1/CTL and Th2/Treg recruitment and polarization.

Figure S3. Expression of cytokines and chemokines in TAMs induced by conditioned medium from PC cells VCaP (VCaP-TaM) and LnCAP (LnCaP-TaM), or CRC cells Caco2 (Caco2-TaM) and HKE3 (HKE3-TaM). Gene expression was analyzed by RT-PCR. Shown is the fold gene expression ± SD with Ctrl macrophages set as 1.

Supplementary Table S1. Cytokine and chemokine expression in differentiated macrophages (Ctrl), PC-TaMs and CRC-TaMs.

Gene symbol	Ctrl	PC-TaM	CRC-TaM
ADIPOQ	1,62E-05	2,26E-05	2,00E-05
BMP2	4,18E-04	8,24E-05	2,00E-05
BMP4	4,15E-05	2,26E-05	2,00E-05
BMP6	1,62E-05	2,26E-05	2,28E-04
BMP7	1,62E-05	2,26E-05	2,00E-05
C5	3,51E-03	3,64E-03	6,83E-03
CCL1	1,62E-05	1,22E-04	7,57E-04
CCL11	6,29E-05	2,33E-04	3,13E-05
CCL13	6,37E-04	7,32E-04	2,30E+00
CCL17	1,62E-05	2,26E-05	1,02E-04
CCL18	6,54E-04	1,74E-03	5,92E-02
CCL19	1,62E-05	2,26E-05	6,48E-05
CCL2	1,52E+0	1,57E+00	2,09E+00
CCL20	1,62E-05	9,94E-05	2,30E-04
CCL21	1,62E-05	2,26E-05	4,97E-05
CCL22	1,71E-04	1,28E-03	4,69E-04
CCL24	1,43E-04	1,35E-04	1,63E-01
CCL3	3,79E-03	1,71E-02	7,73E-03
CCL5	1,18E-03	3,40E-03	4,86E-03
CCL7	1,80E-04	6,93E-04	3,68E-01
CCL8	1,62E-05	2,26E-05	8,04E-05
CD40LG	2,10E-04	2,40E-04	3,19E-04
CNTF	3,03E-03	1,12E-03	2,15E-03
CSF1	2,39E-04	7,01E-04	6,34E-03
CSF2	4,96E-05	2,68E-04	6,67E-05
CSF3	3,79E-04	4,24E-04	4,01E-04
CX3CL1	5,77E-05	1,20E-04	2,00E-05
CXCL1	1,18E-02	2,93E-02	5,69E-02
CXCL10	1,11E-03	4,06E-04	9,40E-04
CXCL11	1,62E-05	2,26E-05	2,00E-05
CXCL12	1,92E-04	1,00E-03	2,00E-05
CXCL13	4,00E-05	2,26E-05	1,13E-04
CXCL16	1,07E-02	3,36E-02	5,87E-02
CXCL2	2,23E-02	8,57E-02	2,31E-01
CXCL5	5,40E-04	6,18E-03	2,95E+00
CXCL9	3,27E-05	3,53E-05	5,58E-05
FASLG	1,62E-05	2,26E-05	2,00E-05
GPI	1,06E-01	1,38E-01	1,62E-01
IFNA2	1,62E-05	2,26E-05	2,00E-05
IFNG	1,62E-05	2,26E-05	2,00E-05
IL10	6,26E-03	7,76E-03	4,54E-03
IL11	1,62E-05	2,26E-05	2,00E-05
IL12A	1,62E-05	1,07E-04	2,00E-05
IL12B	1,62E-05	2,26E-05	9,61E-05
IL13	1,02E-04	1,80E-04	1,94E-04

IL15	1,28E-03	1,20E-03	3,46E-03
IL16	1,84E-02	2,88E-02	2,07E-02
IL17A	1,62E-05	2,26E-05	2,00E-05
IL17F	1,62E-05	2,26E-05	2,00E-05
IL18	9,35E-02	7,55E-02	7,84E-02
IL1A	1,39E-03	3,09E-04	1,73E-03
IL1B	2,11E-03	2,49E-03	1,51E-02
IL1RN	6,22E-03	1,16E-02	1,98E-01
IL2	1,62E-05	2,26E-05	2,00E-05
IL21	4,35E-05	1,12E-04	9,12E-05
IL22	1,09E-04	1,59E-04	2,19E-04
IL23A	5,73E-04	1,99E-04	5,23E-04
IL24	2,07E-05	1,24E-04	1,05E-04
IL27	1,13E-04	1,13E-04	4,14E-04
IL3	1,62E-05	2,26E-05	2,00E-05
IL4	5,82E-05	2,26E-05	2,00E-05
IL5	1,62E-05	2,26E-05	1,47E-04
IL6	1,47E-03	3,72E-04	9,96E-03
IL7	1,03E-03	6,60E-04	4,30E-04
IL8	7,16E-03	9,99E-02	5,29E-01
IL9	2,21E-05	4,11E-05	1,73E-04
LIF	1,55E-04	1,10E-04	2,39E-04
LTA	1,62E-05	2,31E-05	2,00E-05
LTB	5,30E-04	4,96E-04	3,93E-03
MIF	1,71E-01	1,69E-01	3,29E-01
MSTN	2,20E-05	2,95E-05	2,00E-05
NODAL	1,34E-04	8,25E-05	2,38E-04
OSM	4,54E-03	1,98E-03	2,59E-03
PPBP	6,49E-04	2,43E-03	1,75E+01
SPP1	7,30E-01	3,15E-01	1,13E+00
TGFB2	4,22E-03	3,35E-03	5,13E-04
THPO	1,62E-05	2,26E-05	2,00E-05
TNF	9,60E-03	7,12E-03	1,39E-02
TNFRSF11	1,62E-05	2,26E-05	2,14E-05
TNFSF10	1,15E-02	1,25E-02	6,32E-02
TNFSF11	1,56E-04	3,62E-04	9,55E-05
TNFSF13B	3,63E-02	2,74E-02	3,87E-02
VEGFA	4,27E-03	6,79E-03	1,75E-02
XCL1	1,62E-05	2,26E-05	2,00E-05

Normalized expression of cytokine and chemokine genes, according to the 2^ -ΔCt (Ct(GOI) - Ave Ct (HKG)) data analysis method. GOI; Gene of interest, HKG; House keeping gene.
